# Evil Effects of Catarrh

**Published:** 1890-07

**Authors:** 


### EVIL EFFECTS OF CATARRH.

It has been the rule to consider nasal catarrh almost entirely a local disease, and one which has very little effect upon the general system. All this is being rapidly disproved, and it is being shown that if the nose is in an unhealthy state there is quite a long list of affections which may be induced in consequence. Hay fever has often been cured by applications to certain points in the nasal passages. Asthma, also, has yielded to the same treatment. It is a well known fact that when the victim of catarrh suffers from dyspepsia, scarcely any improvement in the latter can be made, no matter what is done for it, until the former is on the gain. Evidence is not wanting to show that severe functional disturbances of the lungs, and even of the heart, are sometimes induced by nasal trouble. Recently, there was reported the case of a woman who had had epilepsy for several years, and although she had patiently sought relief, no improvement took place until applications were made to her nose and its condition improved. Under that line of treatment, entire recovery occurred. Very many other cases are on record which show that the relation between the nose and other parts of the system is very intimate, the connection being, of course, through the nerve system.

